# Chinese Herbal Medicine Alleviates Myocardial Ischemia/Reperfusion Injury by Regulating Endoplasmic Reticulum Stress

**DOI:** 10.1155/2021/4963346

**Published:** 2021-12-07

**Authors:** Wu-Lin Liang, Meng-Ru Cai, Ming-Qian Zhang, Shuang Cui, Tian-Rui Zhang, Wen-Hao Cheng, Yong-Hong Wu, Wen-Jing Ou, Zhan-Hong Jia, Shuo-Feng Zhang

**Affiliations:** ^1^School of Chinese Materia Medica, Beijing University of Chinese Medicine, Beijing 102488, China; ^2^Tibetan Medicine Department of Tibetan Traditional Medical College, Lhasa 850030, China

## Abstract

Myocardial ischemia/reperfusion injury is the main cause of increased mortality and disability in cardiovascular diseases. The injury involves many pathological processes, such as oxidative stress, calcium homeostasis imbalance, inflammation, and energy metabolism disorders, and these pathological stimuli can activate endoplasmic reticulum stress. In the early stage of ischemia, endoplasmic reticulum stress alleviates the injury as an adaptive survival response, but the long-term stress on endoplasmic reticulum amplifies oxidative stress, inflammation, and calcium overload to accelerate cell damage and apoptosis. Therefore, regulation of endoplasmic reticulum stress may be a mechanism to improve ischemia/reperfusion injury. Chinese herbal medicine has a long history of clinical application and unique advantages in the treatment of ischemic heart diseases. This review focuses on the effect of Chinese herbal medicine on myocardial ischemia/reperfusion injury from the perspective of regulation of endoplasmic reticulum stress.

## 1. Introduction

Myocardial infarction continues to be a leading contributor to deaths globally. The timely recovery of blood perfusion to the ischemic myocardium remains the most effective treatment strategy for myocardial infarction, and successful recovery significantly reduces the morbidity and mortality. However, after the resumption of blood flow and reperfusion, myocardial injury can be aggravated, and cardiomyocyte apoptosis can be induced, in a process known as myocardial ischemia/reperfusion (I/R) injury [1].

The endoplasmic reticulum (ER) is a multifunctional organelle in eukaryotic cells. It participates in calcium homeostasis and lipid and cholesterol synthesis, and it is responsible for the synthesis, folding, and assembly of secretory and transmembrane proteins. Approximately 30% of proteins are produced and modified in the ER; this modification process includes protein folding, glycosylation, and disulfide bond formation [2]. Because protein folding is a complex, error-prone process, ER has developed a quality control system. The ER quality control system prevents protein aggregation by promoting correct folding or selective degradation of misfolded peptides, and it is regulated by molecular chaperones, folding enzymes, and lectins [3]. The synthesis of ER proteins requires a highly stable internal environment. However, during myocardial ischemia, the disturbance of oxidative stress, calcium homeostasis imbalance, inflammation, energy metabolism disorders, and more lead to dysfunction in the ER quality control system, resulting in ER stress that is characterized by the accumulation of unfolded and/or misfolded proteins in the ER lumen. Increasing evidence suggests that ER stress is activated in ischemic heart diseases and is widely involved in the pathological process of hypertension, arrhythmia, heart failure, and other cardiovascular diseases (CVDs) [4]. ER stress initiates the unfolded protein response (UPR) and activates the ER-related degradation (ERAD) system to transport misfolded proteins to the cytoplasm for proteasome degradation, thus reducing the protein load and maintaining the protein balance in the ER [5]. As an adaptive survival response, the UPR maintains cell homeostasis by increasing ER-resident chaperone proteins, accelerating unfolded protein degradation, and reducing protein synthesis. However, a prolonged and severe UPR will activate the ER stress-induced apoptosis pathway, which in turn will promote cell injury and apoptosis.

Chinese herbal medicine has a long history of clinical use to treat CVDs and improve myocardial I/R injury by regulating autophagy, oxidative stress, and mitochondrial function [6–8]. Chinese herbal medicine can reduce brain I/R injury by intervening in the ER stress [9], but the mechanism by which Chinese herbal medicine regulates the ER stress to improve myocardial I/R injury is not clear. This review focuses on the roles of ER stress in the pathological process of myocardial I/R and the potential efficacy of Chinese herbal medicine in protecting the myocardium against I/R injury by regulating ER stress and related targets of the UPR.

## 2. Overview of UPR Activated by ER Stress

**Figure 1:**
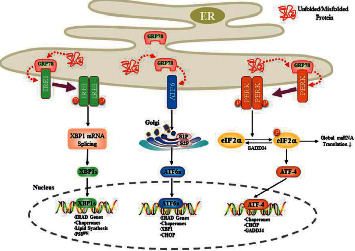
UPR signaling pathway. With ER stress, GRP78 dissociates from three ER transmembrane sensors—IRE1, PERK, and ATF6—to allow their activation. Active IRE1 cleaves the mRNA encoding XBP1 to produce activated XBP1s, which enters the nucleus and induces the transcription of UPR target genes. Active PERK phosphorylates eIF2*α* to inhibit protein synthesis while upregulating ATF4 to active the UPR target genes. ATF4 also induces GADD34 to dephosphorylate eIF2*α*. ATF6 is hydrolyzed in the Golgi to produce an active fragment that enters into the nucleus to induce expression of UPR target genes.

The UPR is activated during ER stress in response to the accumulation of unfolded/misfolded proteins in the ER lumen. The UPR is mediated by a chaperone immunoglobulin/78 kDa glucose regulatory protein (BIP/GRP78) and three transmembrane signal sensors: inositol-requiring kinase 1 (IRE1), protein kinase-like ER kinase (PERK), and activation of transcription factor 6 (ATF6). These sensors are classified as type I and type II transmembrane proteins. IRE1 and PERK are type I transmembrane proteins with protein kinase activity, and ATF6 is a type II transmembrane protein that encodes transcription factors [10]. All three sensors have a lumen domain that binds to the ER chaperone GRP78. Under normal conditions, these three sensors bind to GRP78 and stabilize on the ER membrane; during ER stress, the unfolded/misfolded proteins in the ER lumen compete with the three sensors to bind GRP78, which triggers the corresponding UPR pathways to activate ERAD, reduces protein synthesis, and enhances the protein folding ability (Figure 1) [11].

### 2.1. IRE1-Mediated UPR Signaling

IRE1 is the most elementary ER stress sensor. IRE1 signaling is found throughout the animal kingdom, from lower eukaryotes, such as yeast, to mammals; conversely, PERK and ATF6 are only found in higher animals [12]. Two subtypes of IRE1 exist: IRE1*α* and IRE1*β*. IRE1*α* is expressed in all types of cells, but IRE*β* is only specifically expressed in intestinal epithelial cells [13]. Stimulated by the accumulation of misfolded proteins, IRE1 dimerizes and autophosphorylates. Upon ER stress, the activated IRE1 exhibits endoribonuclease activity, affecting multiple downstream targets. In mammalian cells, IRE1 recognizes and cleaves an intron in the mRNA encoding X-box binding protein (XBP) 1. The cleaved XBP1 (XBP1s) is translated into a more powerful basic leucine zipper (B-ZIP) transcription factor. XBP1s enters the nucleus and binds to the ER stress response element (ESE) in the promoter regions of the UPR target genes to induce the transcription of genes participating in chaperones, protein folding, ERAD, and regulation of metabolism [14]. However, persistent ER stress triggers IRE1 to induce the degradation of certain mRNAs or microRNAs in a process known as regulated IRE1-dependent decay (RIDD). The early stage of RIDD may play a beneficial role in ER homeostasis by reducing the ER protein load, but sustained RIDD degrades some mRNAs encoding survivin and induces cell apoptosis. Long-term activation of IRE1 may lead to the rapid decline of several microRNAs of cysteinyl aspartate specific proteinase (caspase)-2, leading to an increase in the caspase-2 protein level and cell death [15, 16].

### 2.2. PERK-Mediated UPR Signaling

PERK is a transmembrane serine/threonine kinase that is activated via dimerization and autophosphorylation during ER stress. Active PERK phosphorylates eukaryotic translation initiation factor 2 alpha (eIF2*α*), leading to a global inhibition of protein synthesis in the cells [17, 18]. In addition, PERK activation phosphorylates nuclear factor E2 related factor 2 (Nrf2), which is an antioxidant transcription factor that can initiate the transcription of antioxidant genes, such as heme oxygenase 1 (HO-1) and glutathione *S*-transferase [19, 20]. However, in chronic or severe ER stress, PERK-mediated phosphorylation of eIF2*α* promotes the selective translation of several mRNAs, such as those encoding activating transcription factor 4 (ATF4). In mammals, phosphorylated eIF2*α* activates the translation of ATF4, which in turn stimulates the expression of the downstream proapoptotic factor C/EBP homologous protein (CHOP). CHOP regulates a variety of apoptosis-related genes; for example, it inhibits the expression of B-cell lymphoma-2 (Bcl-2) and promotes growth arrest and DNA damage-inducible protein 34 (GADD34) [21]. GADD34 interacts with the catalytic subunit of type I protein serine/threonine phosphatase, thus promoting the dephosphorylation of eIF2*α* in a negative feedback loop [22].

### 2.3. ATF6-Mediated UPR Signaling

ATF6 is a B-ZIP family transcription factor that contains a central transmembrane domain and is located in the ER membrane; it has two subtypes: ATF6*α* and ATF6*β*. ATF6*α* has higher transcriptional activity than ATF6*β*. Under normal conditions, ATF6 is localized on the ER membrane by interacting with GRP78. When the UPR is activated, ATF6 dissociates from GRP78 and transfers from the ER to the Golgi [23, 24]. In the Golgi, ATF6 is hydrolyzed by Site-1 and Site-2 proteases to produce cytoplasmic fragments with transcriptional activity; then, the processed nuclear ATF6 (ATF6n) enters the nucleus and binds to the ESE in the promoter regions of the UPR target genes to promote the transcription of genes involved in protein folding and degradation, thus enhancing the folding ability of the ER and restoring the stability of the intracellular environment [25]. The main downstream targets of ATF6 are chaperones, transcription factor XBP1, and CHOP [26].

In conclusion, three UPR signaling pathways are activated during ER stress. Activation increases the folding ability of the ER protein and decreases protein translation by promoting the transcription of a series of downstream target genes to restore ER homeostasis and promote cell survival. However, prolonged ER stress may transform prosurvival signaling in the UPR into proapoptotic signaling. Therefore, the timing and extent of UPR activation are crucial to promote cell survival and restore cell homeostasis.

## 3. Regulation of Myocardial I/R Injury by ER Stress

**Figure 2:**
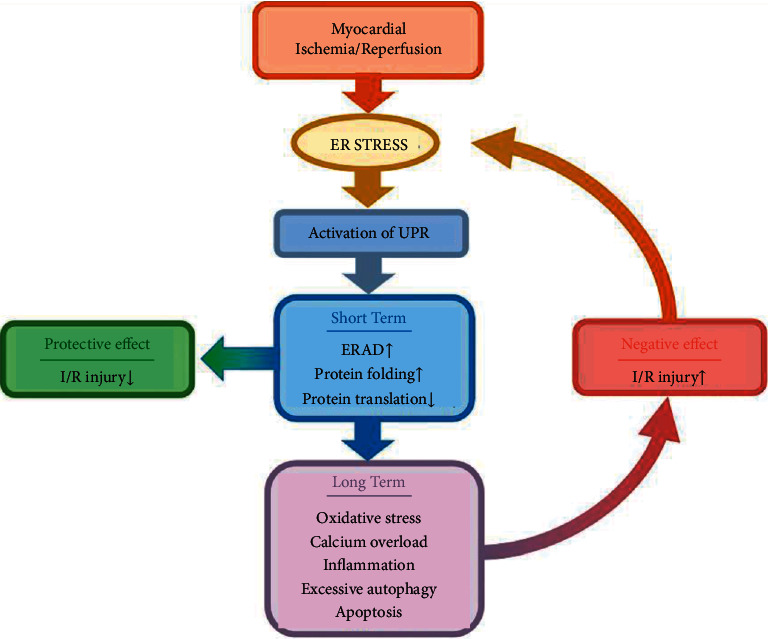
Myocardial I/R injury and ER stress. Myocardial I/R activates ER stress, and ER stress activates the UPR. Short-term UPR restores ER stability by activating ERAD, increasing the ER protein folding ability and inhibiting protein synthesis, which alleviates myocardial I/R injury. However, long-term UPR aggravates myocardial I/R injury by activating oxidative stress, calcium overload, inflammation, excessive autophagy, and apoptosis.

ER stress and the UPR are activated during myocardial I/R and mediate a pathological process in the myocardium [27]. In fact, ER stress can be activated by a variety of pathological factors during I/R, and any one of the pathological stimuli—such as a sudden load of oxygen free radicals, production of proinflammatory cytokines, or calcium homeostasis imbalance—may activate the adaptive survival pathway and the proapoptotic pathway of the UPR. However, during long-term I/R injury, the prosurvival effect of the UPR eventually changes to a proapoptotic effect (Figure 2).

### 3.1. Prosurvival Effect of ER Stress

#### 3.1.1. Effect of IRE1/XBP1 Signaling

IRE1/XBP1 plays an important role in CVDs. In an I/R model established by coronary artery ligation in mice, XBP1s was induced after 5 min of reperfusion and increased approximately 6-fold after 4 h of reperfusion. The downstream target genes of XBP1, such as GRP78 and GRP94, also increased after 4 h of reperfusion [28]. A cardiac-specific XBP1 gene knockout (CKO) animal model has been used to better study the role of the IRE1/XBP1s pathway in the process of myocardial I/R [28]. Compared with the mice in the control group, the mice in the CKO group had increased myocardial infarction size, impaired cardiac function, and increased myocardial hypertrophy. Conversely, the myocardial infarct size and the cardiac impairment of XBP1-overexpressing transgenic mice were reduced after I/R injury, which highlighted the protective effect of XBP1 on cardiomyocytes. These results suggested that XBP1 induction is necessary to protect the myocardium from I/R injury; the potential mechanism might involve transcriptional enhancement of the hexosamine biosynthesis pathway [28]. At the same time, in the brain I/R injury model, XBP1 overexpression also inhibited cell death [29].

#### 3.1.2. Effect of PERK Signaling

Activation of the PERK branch has been observed during myocardial ischemia, and the downstream target eIF2*α* is phosphorylated in the early stages of ischemia [30]. Overexpression of PERK promotes cell survival in hypoxia, and downregulation of PERK leads to decreased cell survival, which may be related to the decreased phosphorylation of eIF2*α* that impairs resistance to misfolded proteins accumulation in the ER, oxidative stress, and nutritional deprivation [31]. However, during oxidative stress associated with ischemia, PERK has been involved in the phosphorylation of Nrf2, which translocates to the nucleus and activates the transcription of antioxidant-related genes [32]. A recent study has shown that PERK has a protective effect on myocardial I/R injury via upregulation of Nrf2/HO-1 signal transduction. Overexpression of Nrf2 and HO-1 improves cardiomyocyte apoptosis and reduces the myocardial infarction area [33]. However, when PERK is activated for a long time, cardiomyocyte death is triggered. In cardiomyocytes, ischemia-induced, persistent ER stress promotes activation of the PERK/ATF4/CHOP axis [34, 35]. Activation of the ATF4/CHOP axis upregulates another effector: tribbles-related protein 3 (TRB3). TRB3 increases during a myocardial infarction, and overexpression of TRB3 in cardiomyocytes in mice has shown that pathological cardiac remodeling and apoptosis increase after myocardial infarction [36, 37]. The persistent activation of PERK triggers an apoptosis signal initiated by ER stress. Thus, PERK signaling may play a negative role in myocardial I/R.

#### 3.1.3. Effect of ATF6 Signaling

ATF6 plays an important and unique role in the heart. ATF6 is activated during ischemia and inactivated during reperfusion, which indicates that ATF6 has a preconditioning effect on cell survival during reperfusion. The expression of GRP78 is stimulated in cardiomyocytes after 20 h of simulated ischemia. When ATF6 is inhibited by siRNA (small interfering RNA), this effect is greatly weakened, and the death of cardiomyocytes during reperfusion increases, indicating that the expression of ATF6 has a protective effect on myocardial reperfusion injury [38]. Transgenic mice models with a tamoxifen-regulated form of ATF6 also have been used to study the role of ATF6 in protecting the myocardium from I/R injury [27]. After induction of ATF6n by tamoxifen, the hearts of transgenic mice showed significant protective effects against I/R, such as increased recovery of ventricular development pressure, decreased cardiomyocyte apoptosis, and decreased lactate dehydrogenase (LDH) release [27]. These results indicate that the ATF6 branch of the UPR has a strong effect on reducing myocardial I/R injury. ATF6 also protects the heart by inducing ERAD, and it promotes the degradation of the terminal misfolded proteins in the ER [39]. The ability to clear the misfolded proteins from the ER is particularly important in the process of ischemic stress. Derlin-3, one of the early components of ERAD, is a reverse translocation channel that is induced by ATF6 in the heart. Overexpression of Derlin-3 enhances the clearance of misfolded proteins, reduces ER stress activation and caspase activity, and protects cardiomyocytes from ischemia-induced apoptosis [39]. Conversely, Derlin-3 knockout attenuates the clearance of misfolded proteins and increases cell death after I/R [39].

A recent study highlighted the new role of ATF6 in protecting the heart from I/R damage. Overproduction of reactive oxygen species (ROS) is the key pathological process of reperfusion injury. The study results showed that overexpression of ATF6n was enough to protect neonatal rat cardiomyocytes (NRCMs) from H_2_O_2_-induced cell damage [40]. ATF6 knockout increased ROS production and cell death after I/R. Additional research showed that two conserved ESE sites existed in the catalase (CAT) promoter region, suggesting that CAT was a direct transcription target of ATF6. ATF6 gene knockout also significantly reduced the expression of CAT after I/R in mice. An in vitro study demonstrated that the lack of ATF6 aggravated the I/R injury and the overexpression of ATF6 or CAT reduced the damage. That study found that ATF6 established a relationship between ER stress and oxidative stress, which provided a new understanding of the treatment of I/R injury [40]. The small-molecule *N*-(2-hydroxy-5-methylphenyl)-3-phenylpropanamide is a drug that activates ATF6 preferentially [41]. A recent study showed that this molecule had a strong protective effect on reperfusion injury of different tissues, including the myocardium, highlighting the therapeutic potential of a cellular proteostasis reprogramming strategy for improving I/R injury [42]. In conclusion, these findings suggest that ATF6 has a strong cardioprotective effect on I/R mediated by a variety of prosurvival mechanisms.

### 3.2. Prodamage Effect of ER Stress

ER stress and ER dysfunction have been induced by increasing the expression of calreticulin (CRT), c-Jun NH2-terminal kinase (JNK), and caspase-12 after myocardial I/R. Ischemic postconditioning protected the myocardium from I/R injury by inhibiting ER stress through the p38 and JNK pathways [43]. ER stress is also involved in myocardial infarction, and inhibition of ER stress can prevent postinfarction-induced cardiac rupture and remodeling by modulating both cardiac apoptosis and fibrosis [44]. These studies indicate that ER stress is activated by I/R injury and that ER stress also increases the myocardial I/R injury by stimulating multiple pathological processes.

#### 3.2.1. ER Stress Promotes Oxidative Stress Injury

Oxidative stress is the primary pathological process during I/R, and the increase of ROS is the main cause of oxidative stress injury. During the process of myocardial I/R, ROS is produced and released [45]. Oxidative stress activates ER stress, which in turn promotes oxidative stress. In many in vitro and in vivo models, ER stress and oxidative stress promote each other, interfering with the normal functions of cells and activating a proapoptotic signal [46].

Approximately 25% of ROS produced in cells comes from the ER, mainly via the folding of oxidized proteins and the formation of disulfide bonds between cysteines [47]. Protein folding is coupled with ROS formation. Protein disulfide isomerase (PDI) and ER oxidoreductase 1 (ERO1) are combined in the ER to produce H_2_O_2_ during catalyzation and formation of disulfide bonds. H_2_O_2_ is the most abundant ROS produced in the ER. During ER stress, the accumulation of misfolded proteins requires more disulfide bond formation and isomerization, leading to more H_2_O_2_; eventually, H_2_O_2_ accumulation exceeds the antioxidant capacity of the ER, resulting in oxidative stress injury [48]. Although disulfide bond formation in the ER is an important source of ROS, protein misfolding in the ER can deplete the glutathione level in the ER and lead to oxidative stress [49].

NADPH oxidase (NOX) is also involved in the production of ROS during ER stress, and the NOX2 and NOX4 subtypes in particular are involved in the regulation of ER stress [50]. NOX2 and NOX4 are the main NOX subtypes in cardiomyocytes; NOX2 is mainly located in the plasma membrane, and NOX4 is mainly located in the mitochondria and the ER membrane [51]. During ER stress, NOX4 may participate in the formation of a highly oxidized ER environment to trigger upstream UPR signals [50]. Calcium/calmodulin-dependent protein kinase II (CaMK II) triggers NOX2-induced oxidative stress, which is related to ER stress-induced apoptosis and renal dysfunction [52].

CHOP, as the main proapoptotic factor of UPR, also induces oxidative stress in different ways. In mammalian cells, ERO1 is transcriptionally activated by CHOP, and ERO1 increases the production of ROS during ER stress [53]. The IRE1 pathway of the UPR activates JNK, which interacts with the mitochondrial JNK binding protein, leading to respiratory dysfunction and an increase in mitochondrial ROS, thus maintaining the activation of JNK and eventually leading to apoptosis [54]. This event links UPR activation with oxidative stress-induced apoptosis. The IRE1 pathway also increases the mRNA level of thioredoxin-interacting protein (TXNIP) by reducing a TXNIP-destabilizing microRNA, miR-17, to generate ROS and participate in the occurrence of oxidative stress [55].

Mitochondria represent another important part of ROS production in the ER stress process. The ER and mitochondria are connected by a mitochondria-associated ER membrane (MAM). Major Ca^2+^ channels, including inositol-1,4,5-triphosphate receptor (IP3R) and voltage-dependent anion channel, are abundant in MAMs [56, 57]. ATP, Ca^2+^, metabolites, and ROS are rapidly transferred from the ER to mitochondria through MAMs [58, 59]. ROS produced by the ER or other sources target the ER calcium channels, leading to calcium release in the ER. In addition, CHOP regulates the expression of ERO1 and stimulates IP3R-mediated release of Ca^2+^ in the ER [60, 61]. The Ca^2+^ released by the ER is rapidly absorbed by the mitochondria through MAMs, resulting in the opening of the permeability transition pore; then, cytochrome c is released from the mitochondrial matrix. The loss of cytochrome c inhibits the electron transport chain complex III and enhances the production of ROS by increasing the ubisemiquinone radical intermediate. In addition, increased Ca^2+^ in the mitochondria stimulates the Krebs cycle dehydrogenase, which increases oxygen consumption and ROS production. Mitochondrial Ca^2+^ also activates nitric oxide synthase, which disrupts the electron transport chain and promotes the production of ROS [46, 62]. Some researchers believe that PERK is related to MAM. PERK is enriched in the membrane of macrophages and helps tighten the contact site between the ER and mitochondria, promoting calcium influx and ROS-mediated mitochondrial apoptosis in chronic ER stress [63, 64].

ROS induced by ER stress lead to the development of CVDs and trigger a variety of secondary ROS, such as hydroxyl free radicals, hypochlorite, and hydrogen peroxide free radicals, which further weakens the function of the ER and aggravates the progression of CVDs [65]. Some small-molecule antioxidants, such as butylhydroxyanisole, can prevent ER stress-induced apoptosis and promote protein folding [66], which also demonstrates the key role of oxidative stress in cell dysfunction associated with protein misfolding.

#### 3.2.2. ER Stress Promotes Calcium Overload

During ER stress, the dynamic balance of ER redox is destroyed. ROS open the ER calcium channels, including IP3R and ryanodine receptor, resulting in Ca^2+^ leakage. Ca^2+^-mediated ROS production in the mitochondria then promotes Ca^2+^ release from the ER. Therefore, the Ca^2+^-mediated increase in ROS and the ROS-modulated upsurge in Ca^2+^ create a self-amplified loop [67, 68]. In addition, CHOP induces the expression of ERO1, activating IP3R-mediated Ca^2+^ release into the cytoplasm and activating CaMK II to induce apoptosis [69–71]. The UPR also induces the expression of a truncated sarcoplasmic/ER Ca^2+^-ATPase (SERCA) and increases the transport of Ca^2+^ to the mitochondria [72]. During myocardial ischemia, the damaged respiratory electron transport chain affects the production of ATP, leading to increased Ca^2+^ efflux and decreased Ca^2+^ uptake by inhibiting the SERCA pump [73]. In addition, the ER protein sigma-1 receptor (Sig-1R) forms a complex with GRP78 in MAMs, and Sig-1R dissociates from GRP78 after Ca^2+^ depletion in the ER; as a result, a Ca^2+^ signal enters the mitochondria through the IP3R for a long time, causing Ca^2+^ overload in the mitochondria [74]. IRE1*α* also plays an important role in inducing apoptosis by interfering with Ca^2+^ homeostasis via IP3R [75].

The production of ROS mediated by Ca^2+^ also promotes the release of Ca^2+^ in the ER and then destroys Ca^2+^-dependent chaperones and ER homeostasis, leading to ER stress. In addition, ROS itself damage the folding of the oxidation protein in the ER. These mechanisms lead to a vicious circle of ER stress and mitochondrial dysfunction: they promote each other and promote apoptosis altogether. Severe ER stress induces mitochondrial Ca^2+^ overload, ROS accumulation, and ATP depletion, thereby activating mitochondrial-dependent apoptosis [76].

XBP1 and ATF6 may mediate the overexpression of GRP94, thus reducing the necrosis of cardiomyocytes caused by calcium overload or ischemia [77]. In an experimental model of myocardial I/R, inhibition of calpain improved ischemic myocardial injury and cardiac function [78], suggesting that regulation of the Ca^2+^ level to restore calcium homeostasis alleviates the I/R injury caused by ER stress.

#### 3.2.3. ER Stress Promotes Inflammatory Injury

Inflammation is related to ER stress and plays an important role in the development of CVDs [79]. Multiple pathological stimuli that activate the UPR, such as excessive production of ROS, calcium overload, and metabolic disorders, can induce inflammation. When the UPR is activated, IRE1 upregulates tumor necrosis factor receptor associated factor 2 (TRAF2) to form an IRE1-TRAF2 complex and recruits inhibitor of nuclear factor kappa-B (I*κ*B) kinase to activate nuclear transcription factor-*κ*B (NF-*κ*B). NF-*κ*B is the main transcriptional regulator of the proinflammatory pathway. The NF-*κ*B dimer released from the cytoplasm (an NF-*κ*B-I*κ*B complex) translocates to the nucleus and activates the expression of inflammatory cytokines [80]. In addition, the IRE1-TRAF2 complex activates JNK, which activates transcription factor activating protein-1, thereby inducing the expression of inflammatory genes [81]. XBP1, the downstream target gene of IRE1, also induces the production of inflammatory cytokines by enhancing the Toll-like receptor (TLR) signal [80].

ATF6 is a regulator between ER stress and proinflammatory pathways. In response to ER stress, the cleavage of ATF6 leads to the transcriptional activation of inflammation-related proteins, such as C-reactive protein. C-reactive protein participates in the inflammatory response by promoting the expression of monocyte chemoattractant protein-1 (MCP-1) receptors [80, 82]. In addition, the ATF6 pathway activates NF-*κ*B through Akt kinase phosphorylation [83]. As a response to stress, attenuation of I*κ*B via PERK/eIF2*α*-mediated translation leads to NF-*κ*B release and activation; activated NF-*κ*B shifts to the nucleus and triggers the expression of various inflammatory cytokines [84].

CHOP activates interleukin (IL)-1*β*, and CHOP is activated by three ER stress sensors [85]. I/R causes the activation of CHOP and the release of proinflammatory cytokines, such as IL-1*β* and IL-6, which leads to tissue inflammation [35]. In the heart of mice with MCP-1 overexpression, a group of ER stress-related genes (GRP78, GRP94, ATF6, and CHOP) and the ER chaperone PDI were transcriptionally activated, which recruited inflammatory cells into the heart and aggravated the myocardial ischemia injury [86].

ER stress activates nucleotide-binding oligomerization domain-like receptor protein 1 (NLRP1) and promotes myocardial I/R injury by activating the NF-*κ*B signaling pathway [87]. NLRPs are classified as typical inflammasomes, and they include NLRP1 and NLRP3. They activate caspase-1, leading to increases in the levels of the proinflammatory cytokines IL-1*β* and IL-18 [88]. At the same time, a variety of cytokines in the inflammatory response may directly stimulate the UPR [89, 90]. In conclusion, ER stress is closely related to inflammation, and well-consolidated crosstalk exists between ER stress and inflammatory molecules, which may play a key role in the pathogenesis of myocardial I/R injury.

#### 3.2.4. ER Stress Promotes Excessive Autophagy

ER stress is also related to autophagy. Autophagy is activated to degrade misfolded proteins and provide protection for cells. However, persistent ER stress promotes cell death through autophagy [91, 92]. Autophagosomes increase significantly in the heart during I/R injury, and excessive autophagy aggravates myocardial injury [93].

JNK activation plays an important role in the expression of Bcl-2. Bcl-2 is located in the mitochondria, the ER, and the nuclear membrane, and a small amount exists in the cytoplasm [94]. Bcl-2 is an antiapoptotic protein and an autophagy inhibitor [95]. Bcl-2 located in the ER dimerizes with Beclin1, which prevents the formation of autophagosomes. JNK-mediated Bcl-2 phosphorylation interferes with the dimerization of Beclin1 and Bcl-2 and triggers autophagy [96]. In addition, ER stress directly induces autophagy via the ATF4 pathway [97].

Although autophagy is a homeostasis mechanism that provides energy for the damaged heart, uncontrolled excessive autophagy may lead to cell dysfunction or death. In myocardial I/R-induced excessive ER stress, a persistent UPR and the decreased expression of Bcl-2 trigger excessive autophagy [6].

#### 3.2.5. ER Stress Promotes Apoptosis

Apoptosis is a programmed cell death and is regulated by multiple signal pathways. Overexpression of the UPR caused by persistent ER stress will eventually induce apoptosis. It has been confirmed that hypoxia upregulates the expression of CHOP and the cleavage of caspase-12, indicating that the UPR apoptosis pathway is involved in cell death via hypoxia stimulation [98]. In addition, inhibition of CHOP has attenuated myocardial I/R injury in mice by reducing inflammation and apoptosis [35]. ER stress-induced oxidative stress, inflammation, calcium overload, and other pathological processes also accelerate the promotion of apoptosis by activating CHOP, caspase family members, apoptosis signal-regulating kinase 1 (ASK1), JNK, and Bcl-2 family members.

*(1) CHOP Is Activated*. In vitro and in vivo studies have shown that CHOP is the main regulator of ER stress-induced apoptosis. CHOP plays a proapoptotic role in a variety of ischemic conditions [21]. CHOP expression is lower in nonstress conditions, and its expression increases significantly in ER stress through induction of IRE1, PERK, and ATF6-dependent transcription. ATF4 may play a leading role in inducing CHOP expression [69]. IRE1 activates JNK and p38 by interacting with TRAF2 and ASK1. JNK induces cell death by inducing Fas- and NOX2-triggered oxidative stress, whereas p38 activates CHOP by phosphorylation of its transactivation domain [69, 99]. In addition to the abovementioned activation pathways, CHOP is also activated by ATF2, in a process induced by hypoxia and essential to induce CHOP production via leucine starvation [100].

CHOP-induced apoptosis involves many mechanisms. Persistent ER stress activates the expression of death receptor DR5 induced by CHOP, and TNF-related apoptosis-inducing ligand combines with DR5 to activate caspase-8, thus promoting exogenous apoptosis [101]. CHOP inhibits the expression of the antiapoptotic protein Bcl-2 in cardiac myocytes [102, 103]. CHOP also upregulates the expression of the BH3-only domain protein p53 upregulated modulator of apoptosis (PUMA) and Bcl-2 interacting mediator of cell death (BIM), thus inducing mitochondria-dependent apoptosis [104, 105]. In addition, during ER stress, BIM is transferred from a dynein-rich compartment to the ER membrane to activate caspase-12, thus activating an ER stress-specific apoptotic pathway [106]. CHOP-induced apoptosis also involves the calcium signaling pathway. CHOP induces the expression of ERO1, activating IP3R-mediated Ca^2+^ release into the cytoplasm [70]. Intracellular calcium activates CaMK II, which induces the activation of many downstream apoptotic signals, such as the expression of Fas death receptor, activation of JNK, and release of the mitochondrial apoptosis factor [71]. Calcium released from the ER also enters the mitochondria, which induces mitochondria-dependent apoptosis through the release of cytochrome c and loss of the mitochondrial membrane potential [107].

A recent study has shown that CHOP plays an important role in promoting protein synthesis that leads to apoptosis via oxidative stress. The interaction between ATF4 and CHOP directly induces genes that encode protein synthesis and the UPR. Forced expression of ATF4 and CHOP increases protein synthesis but leads to ATP depletion, oxidative stress, and cell death. One of the mechanisms of ATF4/CHOP-stimulated protein synthesis is to activate the transcription of GADD34, leading to the dephosphorylation of eIF2 mRNA to restore global translation. However, in the case of defective protein folding, increased protein synthesis will lead to more misfolding and further aggravate the cell death signal through oxidative stress [108, 109].

Expression of the UPR in retinal ganglion cells has been upregulated by intraocular I/R, whereas the absence of CHOP significantly increased cell survival and improved functional recovery after I/R [110]. Myocardial I/R has activated the phosphorylation of eIF2*α* and upregulated the expression of CHOP, whereas cardiac function was improved and myocardial inflammation was reduced in CHOP-knockout mice after I/R [35]. These findings suggest that inhibition of CHOP is of great significance in reducing I/R injury.

### 3.3. Caspase Family Members Are Activated

The activation of caspase family is a key process of apoptosis induced by ER stress. The activation of caspases-2, -3, -4, -6, -7, -8, -9, and -12 in different in vitro and in vivo models of ER stress has been reported. The deletion of caspases-3, -7, -9, or -12 has a protective effect on ER stress-induced cell death [111].

Caspase-12 is specifically expressed in the ER stress-induced apoptosis pathway [112]. The precursor of caspase-12 is procaspase-12, which is located on the ER membrane alone or combined with TRAF2. Severe ER stress results in the dissociation and activation of procaspase-12 from the ER membrane [113, 114]. BIM translocation to the ER surface activates caspase-12; activation is also regulated by other pathways. For example, during ER stress, the Ca^2+^ increase in the cytoplasm leads to activation of calpain and its translocation to the ER, where procaspase is cleaved to form caspase-12 [115]. In addition, activated IRE1 recruits TRAF2 to form an IRE1-TRAF2 complex that results in the dissociation of TRAF2 from a TRAF2-procaspase-12 complex and the activation of caspase-12 [113]. Activated caspase-12 starts a positive feedback cycle to activate caspase-3 by activating caspase-9, thus initiating apoptosis [114].

In the process of ER stress, RIDD can reduce the folding load of the ER proteins and restore ER homeostasis. However, in the long-term ER stress process, this process may lead to cell death by degrading the mRNA that encodes survivin [69, 111, 116]. The sustained activation of IRE1*α* has been shown to degrade the rapid attenuation of some microRNAs that inhibit the translation of caspase-2 mRNA, thereby promoting caspase-2-dependent apoptosis during ER stress [15].

### 3.4. Proapoptotic Proteins of Bcl-2 Family Are Activated

The Bcl-2 family members are classified into antiapoptotic proteins and proapoptotic proteins. Antiapoptotic proteins mainly include Bcl-2 and Bcl-xl, whereas proapoptotic proteins are divided into multidomain proteins and BH3-only proteins. Multidomain proteins include BAK and BAX, and BH3-only proteins include BID, BIM, BAD, BIK, and PUMA [117]. Bcl-2 family proteins are also integrated on the ER membrane, thereby regulating Ca^2+^ homeostasis in the ER and ER stress-induced cell death [118, 119]. In response to ER stress, the proapoptotic proteins BAK and BAX undergo conformational changes on the ER membrane, thereby releasing calcium into the cytoplasm and activating the apoptotic signal [107]. BH3-only proteins sense stress and activate multidomain proteins, such as BAX and BAK, leading to increased mitochondrial outer membrane permeability and cytochrome c release, which in turn initiate the caspase cascade and activate the mitochondrial apoptotic pathway. However, Bcl-2 and Bcl-xl antagonize this process [120]. BH3-only proteins mainly regulate apoptosis by inhibiting the expression of Bcl-2 or promoting the expression of multidomain proteins, such as BAK and BAX [121]. In cardiomyocytes, the Bcl-2 family protein NIX, which is located in both the ER and the mitochondrial membrane, induces apoptosis by coordinating with BAX and BAK to regulate calcium in the ER [120]. CHOP-induced increases of PUMA and BIM promote the activation of BAK and BAX to activate mitochondria-dependent apoptosis [122]. In addition, BAX and BAK directly bind to the cytoplasmic domain of IRE1*α* and induce activation of the IRE1*α* signaling pathway [123].

In summary, inhibiting the expression of the proapoptotic proteins of the Bcl-2 family plays an important role in reducing I/R-induced cardiomyocyte death. In ER stress, overexpression of PUMA has induced cardiomyocyte apoptosis, and inhibition of PUMA expression has improved I/R injury in vitro and in vivo [124].

### 3.5. ASK1 and JNK Are Activated

ASK1 is a key regulator of cardiomyocyte apoptosis. A small-molecule inhibitor of ASK1 reduced cardiomyocyte apoptosis and infarct size in a rat model of I/R [125]. ASK1 is related to apoptosis, as induced by tumor necrosis factor family receptor signaling. During ER stress, the IRE1-TRAF2 complex activates ASK1, which further activates JNK and p38. An ASK1-knockout experiment showed that JNK activation and cell death need this kinase [126]. The PERK pathway also activates the JNK proapoptotic pathway [127]. Both JNK and p38 induce apoptosis by participating in the activation of CHOP [99, 128]. JNK inhibits the expression of the antiapoptotic factor Bcl-2, thus inhibiting its antiapoptotic function of regulating calcium flow in the ER and promoting proapoptotic Bcl-2 family members, such as BAX and BAK. JNK plays an important role in apoptosis by stimulating proapoptotic proteins (BAX and BAK) and inactivating the antiapoptotic protein Bcl-2 [129, 130].

## 4. Modulatory Role of Chinese Herbal Medicine in ER Stress in Myocardial I/R

### 4.1. Extractive Compounds of Chinese Herbal Medicine

Extracts refer to the effective components extracted from Chinese herbal medicine. The study of extracts can clarify the possible active components in Chinese herbal medicine and the interaction between them. According to the current experimental studies on extracts of Chinese herbal medicine, the protective effects and mechanisms of these traditional Chinese medicines in myocardial I/R injury may be related to the regulation of ER stress (Table 1).

#### 4.1.1. *Rhizoma coptidis*

Berberine (BBR) is an isoquinoline alkaloid isolated from *Rhizoma coptidis*, which is widely used in the clinic because of its various pharmacological effects [145]. BBR has directly downregulated the phosphorylation of PERK and eIF2*α*, thus inhibiting the expression of ATF4 and CHOP in myocardial tissue to inhibit ER stress induced by I/R. In vivo (model of I/R in rats that underwent operation) and in vitro (H9c2 cells exposed to a simulated I/R environment), BBR has reduced myocardial infarct size, improved cardiac function, and inhibited cardiomyocyte apoptosis and oxidative damage, and its mechanism may be related to activation of the Janus kinase 2/signal transducer and activator of transcription 3 (JAK2/STAT3) pathway [131].

#### 4.1.2. *Schisandra chinensis*

Schisandrin B (SCH B) is the most active and abundant component isolated from *Schisandra chinensis* [146]. Previous studies have shown that SCH B has antioxidant, anti-inflammatory, antitumor, cardioprotective, and neuroprotective effects [147]. SCH B has reduced the infarct size and the serum levels of creatine kinase (CK), LDH, and malondialdehyde (MDA); inhibited the expression of phosphorylated PERK (p-PERK), ATF6, and CHOP in myocardial tissue; and upregulated Bcl-2 and downregulated BAX, caspase-3, and caspase-9 [132]. These actions indicate that SCH B can inhibit the ATF6 and PERK pathways to reduce apoptosis induced by ER stress and so protect against myocardial I/R injury.

#### 4.1.3. *Panax notoginseng*

*Panax notoginseng* has a long history of clinical use, including extensive use in the prevention and treatment of CVDs and cerebrovascular diseases [148]. Notoginsenoside R1 (NgR1) is a new saponin isolated from *Panax notoginseng*. It has pharmacological effects on myocardial infarction, ischemic stroke, acute kidney injury, intestinal injury, and other diseases [149]. NgR1 has improved the cardiac function of isolated hearts in rats after I/R injury, reduced the expression levels of GRP78, p-PERK, and ATF6, and phosphorylated IRE1 (p-IRE1) in H9c2 cardiomyocytes after hypoxia/reoxygenation (H/R). NgR1 also inhibited the expression of proapoptotic proteins, such as CHOP, caspase-12, and phosphorylated JNK (p-JNK). In addition, NgR1 has scavenged free radicals, improved the activity of antioxidant enzymes, and inhibited tunicamycin-induced cell death and cardiac dysfunction; these findings indicate that oxidative stress and ER stress are involved in the protective effect of NgR1 [133].

#### 4.1.4. *Curcuma longa*

Curcumin (CUR) is a kind of polyphenol extracted from *Curcuma longa*; it has anticancer, antibacterial, anti-inflammatory, and antiaging activities [150]. CUR has minimized the death of H9c2 cardiomyocytes induced by H/R in one study [134]. Compared with the control group, the H/R group experienced increases in the levels of LDH and MDA and decreases in superoxide dismutase (SOD) activity. After CUR treatment, the levels of LDH, MDA, and SOD were reversed; the expressions of GRP78 and CHOP and the phosphorylation levels of ERK1/2, p38, and JNK were decreased. These results suggested that CUR protected cardiomyocytes by inhibiting ER stress and the mitogen-activated protein kinase (MAPK) pathway.

#### 4.1.5. *Panax quinquefolium*

*Panax quinquefolium* is a *Panax* plant of the Acanthopanax family, which originated from the northern United States and southern Canada. *Panax quinquefolium* has a tonic effect; its root is used as a Chinese herbal medicine. *Panax quinquefolium* saponins (PQS) have many pharmacological effects, including antianxiety, neuroprotective, cardioprotective, anticancer, and antibacterial activities [151]. A study identified a protective effect of PQS on myocardial I/R injury and H/R-induced cardiomyocyte apoptosis; in that study, PQS also inhibited the excessive ER stress induced by H/R, which was manifested by decreased activation of caspase-12 and decreased expression of GRP78 and CHOP [135]. These results suggest that PQS could reduce the H/R damage of cardiomyocytes via a mechanism related to the inhibition of excessive ER stress.

#### 4.1.6. *Salvia miltiorrhiza*

*Salvia miltiorrhiza* is a widely used Chinese herbal medicine that has a cardioprotective effect in the development of thrombosis, atherosclerosis, and myocardial I/R [152]. Protocatechualdehyde (PCA) is the main bioactive polyphenol in *Salvia miltiorrhiza* [153], and it can protect the brain from oxidative damage during I/R [154]. A recent study pointed out the therapeutic potential of PCA for CVDs [136]. PCA had a protective effect on H9c2 cardiomyocytes and NRCMs induced by oxygen-glucose deprivation/reperfusion (OGD/R). PCA could inhibit the expression of BAX and cleaved caspase-3 in H9c2 cardiomyocytes. Additional results of ER-specific DiOC6(3) and ER-Tracker Red staining showed that PCA could reduce the stress injury in the ER; and mechanistic substudy demonstrated that PCA could inhibit CHOP, GRP78, p-PERK, ATF6, and p-IRE1*α* after OGD/R. Together, these results suggest that PCA inhibits ER stress by regulating the PERK, ATF6, and IRE1*α* pathways.

#### 4.1.7. *Rhodiola rosea* L

Salidroside is the main component of the Chinese herbal medicine *Rhodiola rosea* L. A previous study has shown that salidroside can reduce myocardial ischemia injury induced by isoproterenol by inhibiting the inflammatory signaling pathway [155]. A recent study reported that salidroside could reduce H/R-induced H9c2 cardiomyocyte damage, improve cell survival, reduce LDH release, and reduce ER stress, as represented by the downregulation of CHOP protein expression. In addition, salidroside could alleviate ER stress-induced apoptosis, as manifested by the decreased expression of cleaved caspase-12 and BAX proteins, decreased caspase-3 activity, and increased Bcl-2 expression. Another study showed the decreased expression of p-PERK and p-IRE1*α*, indicating that salidroside could protect H9c2 cardiomyocytes from H/R injury and reduce ER stress or ER stress-induced apoptosis by regulating the expression of PERK and IRE1*α* [137].

#### 4.1.8. *Radix astragali*

Astragaloside IV (AS-IV) is one of the main effective components of *Radix astragali*. Studies have shown that AS-IV has a therapeutic effect on heart failure by promoting angiogenesis, improving energy metabolism, and inhibiting myocardial hypertrophy and fibrosis [156]. In a recent study, AS-IV significantly improved the pathological changes of myocardial tissue in a rat model of myocardial I/R, inhibiting cardiomyocyte apoptosis; downregulating the expression of CK in myocardial tissue; upregulating the expression of the SERCA 2a protein; and downregulating the expression of GRP78, ATF6, and p-PERK in a dose-dependent manner [138]. These findings indicate that AS-IV can mediate the SERCA 2a signaling pathway and inhibit ER stress to reduce myocardial I/R injury.

#### 4.1.9. *Aralia elata*

*Aralia elata* is widely distributed in Northeast China, East Russia, Japan, and Korea. Its roots and bark can be used as a Chinese herbal medicine; they play a therapeutic role in arrhythmia, arthritis, hypotension, and diabetes [157]. Recently, total saponins from *Aralia elata* significantly reduced the infarct size of rats in an I/R model; improved the myocardial pathological process; reduced the levels of LDH, CK, and MDA; increased the content of SOD; and recovered the activities of Ca^2+^-Mg^2+^-ATPase, Na^+^-K^+^-ATPase, SERCA, and calcineurin. The total saponins also significantly downregulated the expression of GRP78, CHOP, and BAX and upregulated the expression of Bcl-2, indicating that they can prevent myocardial I/R injury, alleviate the calcium homeostasis imbalance, and reduce ER stress-induced apoptosis [139]. Another study using *Aralia elata* showed that *Aralia* saponin C (AsC) could significantly inhibit H/R injury in H9c2 cardiomyocytes; other effects included increasing cell viability, reducing LDH leakage, and preventing cardiomyocyte apoptosis. AsC also suppressed H/R-induced ER stress by reducing activation of the ER stress pathways (PERK/eIF2*α* and ATF6) and reducing the expression of ER stress-related apoptotic proteins (CHOP and caspase-12). Compared with an H/R group, the AsC treatment group had a significantly increased expression of heat shock protein 90 (HSP90). HSP90 inhibitors and HSP90 siRNA could block the therapeutic effect of AsC, indicating that AsC may attenuate the ER stress-dependent apoptotic pathway by increasing the expression of HSP90, thereby reducing H/R-induced apoptosis [140]. In addition, elatoside C, a triterpenoid component of *Aralia elata*, had a significant protective effect on the death of cardiomyocytes induced by H/R [141]. Elatoside C could maintain the mitochondrial membrane potential as well as reduce mitochondrial ROS and apoptosis; its action is related to the inhibition of ER stress-related apoptosis markers (GRP78, CHOP, caspase-12, and JNK), increased phosphorylation of STAT3, and an increased Bcl-2/BAX ratio. However, these effects of elatoside C could be blocked by STAT3 inhibitors. These results suggest that elatoside C may reduce H/R-induced cardiomyocyte apoptosis by activating the STAT3 pathway and reducing ER stress-related apoptosis.

#### 4.1.10. Other Extractive Compounds

Tournefolic acid B (TAB) in *Clinopodium chinense* (Benth.) Kuntze can significantly improve the hemodynamic parameters of the isolated rat hearts and inhibits cardiomyocyte apoptosis [142]. In addition, TAB inhibits oxidative stress by regulating the activities of antioxidant enzymes and inhibits the expression of GRP78, ATF6, p-PERK, eIF2*α*, CHOP, and caspase-12 to reduce I/R-induced ER stress [142]. TAB also enhances the phosphorylation of phosphoinositide 3-kinase (PI3K) and protein kinase B (Akt), reduces the phosphorylation of JNK, and increases the ratio of Bcl-2 to BAX, indicating that TAB may inhibit PI3K/Akt-mediated ER stress, oxidative stress, and apoptosis to reduce myocardial I/R injury [142].

Resveratrol is a natural polyphenol with various pharmacological effects, including anti-inflammatory, antioxidant, anticancer, and antiapoptotic effects. It exists in the traditional Chinese medicine mulberry and in other plants [158]. Resveratrol has reduced the area of myocardial infarction and lowered serum cardiac troponin I (cTnI) level in a rat model of I/R. It also has inhibited GRP78, TRAF2, Beclin-1, and microtubule-associated protein 1 light chain 3 (LC3) II/I protein expression. Resveratrol has also significantly increased H/R-induced myocardial cell viability, inhibited apoptosis, and reduced the intracellular Ca^2+^ concentration, which all suggest that resveratrol can reduce myocardial I/R injury by inhibiting oxidative stress, ER stress, autophagy, and Ca^2+^ influx [143].

The pharmacological effects of aloe vera on immunity as well as its anti-inflammatory and antioxidant effects have been confirmed [159]. Barbaloin (BAR), the main active ingredient in aloe vera, has attracted attention [160]. A recent study in a rat model of I/R has shown that BAR can reduce myocardial injury and apoptosis and can inhibit the expression of ER stress-related proteins (GRP78, CHOP, caspases-3/12, p-PERK, and ATF4) in myocardial tissue. The study also showed that canopy homolog 2 (CNPY2) is present in cardiomyocytes and is involved in the development of myocardial I/R by activating the PERK/CHOP signaling pathway. BAR may reduce myocardial I/R injury by inhibiting the apoptosis pathway of CNPY2/PERK [144].

### 4.2. Patented Drugs from Chinese Herbs

Although single Chinese medicine treatments have been proven effective in regulating ER stress induced by myocardial I/R, a combination of multiple Chinese medicines is usually used in clinical practice. Patented drugs from Chinese herbs incorporate a certain combination of Chinese medicines under the guidance of Chinese medicine theory, which finds the best treatment method for diseases and exerts the maximum therapeutic effect of Chinese medicines (Table 2).

Qishen granule (QSG) is composed of four Chinese herbs (*Angelica sinensis*, *Lonicera japonica* Thunb., *Scrophularia ningpoensis* Hemsl., and *Glycyrrhiza uralensis* Fisch.). A previous study has shown that QSG can inhibit myocardial inflammation and fibrosis caused by heart failure through the STAT3 and NF-*κ*B pathways [163]. A recent study reported that QSG can improve cardiac function in rats with myocardial ischemia, reduce inflammatory cell infiltration, and reduce cardiomyocyte apoptosis. In a model of OGD/R-induced H9c2 injury, QSG regulated apoptosis-related proteins to exert antiapoptotic effects, including upregulation of Bcl-2 expression and downregulation of BAX and caspases-3 and -12 expression. The potential mechanism is related to activation of the IRE1-*α-*crystallin B (CryAB) signaling pathway [161].

Shuxuening injection (SXNI) is derived from *Ginkgo biloba* extract, and its active ingredients are mainly flavonoids—ginkgolides and bilobalide. It is used clinically to treat coronary heart disease, angina pectoris, cerebral embolism, and cerebral vasospasm [164]. SXNI has reduced the infarct size and decreased the myocardial enzyme and cTnI levels to reduce the degree of myocardial injury [162]. In addition, SXNI has increased the activities of CAT and glutathione peroxidase (GSH-Px) and decreased the expression levels of MDA, GRP78, CRT, CHOP, and caspase-12 [162]. SXNI has also decreased the serum levels of inflammatory cytokines and reduced TLR4/NF-*κ*B expression, which suggests the protective effect of SXNI on I/R injury acts via regulation of TLR4/NF-*κ*B and reduction of the inflammatory response by inhibiting oxidative stress and ER stress pathways [162].

**Figure 3:**
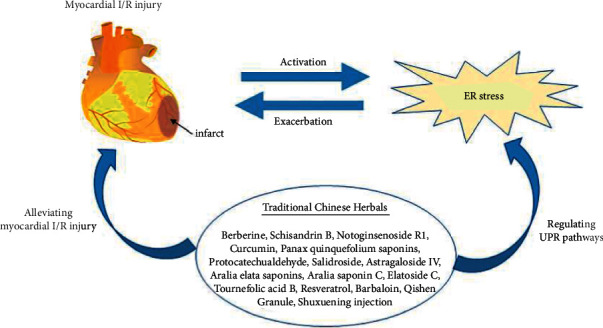
Chinese herbal medicines regulate ER stress to improve myocardial I/R injury.

In conclusion, most Chinese herbal medicines can inhibit the UPR-related signaling pathways to inhibit oxidative stress injury and apoptosis induced by excessive ER stress in myocardial I/R. A few Chinese herbal medicines, such as QSG, can activate the IRE1 signaling pathway and initiate an adaptive UPR to play a protective role in the myocardium. The UPR is activated in the early stage of ischemia and plays a role in promoting survival. After reperfusion, the UPR is continuously activated via the stimulation of oxidative stress, calcium overload, inflammation, and other pathological factors, thus inducing ER stress-mediated apoptosis. Some traditional Chinese medicines can activate the UPR during myocardial ischemia to play a protective role in the myocardium. For example, in mice with ischemic injury, ginkgolide can reduce the size of the myocardial infarction and improve ER dilation [165]. The mechanism is related to an increase in ERAD-mediated clearance of misfolded proteins and autophagy via enhancement of IRE1*α*/XBP1 activity [165]. These studies show that Chinese herbal medicines, through regulation of the UPR in different states of the body, have good application prospects for reducing myocardial I/R injury (Figure 3).

## 5. Conclusions

ER stress is involved in many pathological processes of CVDs, and the UPR activated by ER stress plays a key role. The UPR is a defense mechanism that is induced by three pathways, and it protects cardiomyocytes by maintaining ER homeostasis. However, long-term ER stress leads to cardiomyocyte dysfunction and apoptosis, thereby aggravating damage in CVDs. In myocardial I/R injury, ER stress is activated by multiple pathological processes, such as oxidative stress, inflammation, and calcium homeostasis imbalance. ER stress in turn aggravates these pathological processes, thus forming a vicious circle. Therefore, a deep understanding of the molecular mechanism of ER stress in CVDs is of great significance for the development of new therapeutic methods and the discovery of potential drug targets.

Given the complex signaling network regulation of ER stress, it is important to develop drugs that can act on multiple targets and pathways. Chinese herbal medicine has the characteristics of multiple components, multiple targets, and multiple links and therefore shows great therapeutic potential. In fact, many Chinese medicines, including extracts and patented drugs from Chinese herbs, have improved I/R injury by regulating the ER. However, the exact mechanism by which these Chinese herbal medicines regulate ER stress to improve I/R injury is not clear enough; the period of myocardial ischemia and the degree of myocardial injury must be assessed, and most studies have been carried out in animal and cell models that do not fully align with actual clinical situations. Therefore, additional research is needed to verify the efficacy and mechanisms of Chinese herbal medicines in regulating ER stress to improve I/R injury.

## Figures and Tables

**Table 1:** The role of Chinese herbal extracts in the regulation of ER stress in myocardial I/R.

Compounds	Experimental models	Effects	Mechanisms	References
Berberine	I/R injury in rats and H9c2 cells	Cardiac function↑, myocardial infarct size↓, oxidative damage↓, apoptosis↓, ER stress↓	LDH↓, CK↓, MDA↓, SOD↑, caspase-3↓, Bcl-2/BAX↑, PERK/eIF2*α*↓, ATF4↓, CHOP↓, JAK2/STAT3↑	[131]
Schisandrin B	I/R injury in rats	Myocardial infarct size↓, oxidative damage↓, ER stress↓	LDH↓, CK↓, MDA↓, T-SOD↑, caspases-3/9↓, Bcl-2/BAX↑, p-PERK↓, ATF6↓, CHOP↓	[132]
Notoginsenoside R1	Ex vivo I/R injury model and H/R in H9c2 cells	Cardiac function↑, apoptosis↓, oxidative damage↓, ER stress↓	CK↓, MDA↓, SOD↑, CAT↑, GSH-Px↑, CHOP↓, caspase-12↓, p-JNK↓, GRP78↓, p-PERK↓, ATF6↓, IRE1↓	[133]
Curcumin	H/R in H9c2 cells	Apoptosis,↓ oxidative damage↓, ER stress↓	LDH↓, MDA↓, SOD↑, GRP78↓, CHOP↓, MAPK↓	[134]
*Panax quinquefolium* saponins	H/R in NRCMs	Apoptosis↓, ER stress↓	LDH↓, Bcl-2/BAX↑, caspase-12↓, GRP78↓, CHOP↓	[135]
Protocatechualdehyde	OGD/R in H9c2 cells and NRCMs	Apoptosis↓, ER stress↓	BAX↓, caspase-3↓, GRP78↓, CHOP↓, PERK/ATF6/IRE1*α*↓	[136]
Salidroside	H/R in H9c2 cells	Apoptosis↓, ER stress↓	LDH↓, CHOP↓, caspases-3/12↓, Bcl-2/BAX↑, p-PERK↑ p-IRE1*α*↑	[137]
Astragaloside IV	I/R injury in rats	Apoptosis↓, ER stress↓	CK↓, GRP78↓, ATF6↓, p-PERK↓, SERCA 2a↑	[138]
Total saponins of *Aralia elata*	I/R injury in rats	Myocardial infarct size↓, oxidative damage↓, calcium overload↓, ER stress↓	LDH↓, CK↓, MDA↓,SOD↑, Ca^2+^-Mg^2+^-ATPase↑, Na^+^-K^+^-ATPase↑, SERCA↑, CaN↑, Bcl-2/BAX↑, GRP78↓, CHOP↓	[139]
*Aralia* saponin C	H/R in H9c2 cells	Apoptosis↓, ER stress↓	LDH↓, PERK/eIF2*α*↓, ATF6↓, caspase-12↓, HSP90↑	[140]
Elatoside C	H/R in H9c2 cells	Oxidative damage↓, apoptosis↓, ER stress↓	ROS↓, Bcl-2/BAX↑, GRP78↓, CHOP↓, caspase-12↓, p-JNK↓, STAT3↑	[141]
Tournefolic acid B	Ex vivo I/R injury model and H/R in H9c2 cells	Cardiac function↑, oxidative damage↓, apoptosis↓, ER stress↓	SOD↑, CAT↑, GSH-Px↑, Bcl-2/BAX↑, GRP78↓, CHOP↓, caspase-12↓, ATF6↓, PERK/eIf2*α*↓, p-JNK↓, PI3K/AKT↑	[142]
Resveratrol	I/R injury in rats and NRCMs	Myocardial infarct size↓, oxidative damage↓, autophagy↓, calcium overload↓, ER stress	ROS↓, MDA↓, cTnI↓ Beclin-1↓, LC3 II/I↓, Ca^2+^↓, GRP78↓	[143]
Barbaloin	I/R injury in rats	Apoptosis↓, ER stress↓	Caspases-3/12↓, GRP78↓, CHOP↓, ATF4↓, CNPY2/PERK↓	[144]

**Table 2:** The role of patented drugs from Chinese herbs in the regulation of ER stress in myocardial I/R.

Patented drugs	Experimental models	Effects	Mechanisms	References
Qishen granule	OGD/R in H9c2 cells	Apoptosis↓, ER stress↓	Bcl-2/BAX↑, caspases-3/12↓, IRE1-CryAB↑	[161]
Shuxuening injection	I/R injury in rats	Oxidative damage↓, inflammation↓, ER stress↓	CK↓, LDH↓, cTnI↓, MDA↓, SOD↑, GSH-Px↑, CAT↑, GRP78↓, CRT↓, CHOP↓, caspase-12↓, TLR4/NF-*κ*B↓	[162]

## References

[1] Kalogeris T., Baines C. P., Krenz M., Korthuis R. J. (2016). Ischemia/reperfusion. *Comprehensive Physiology*.

[2] Almanza A., Carlesso A., Chintha C. (2019). Endoplasmic reticulum stress signalling—from basic mechanisms to clinical applications. *FEBS Journal*.

[3] Liu M.-q., Chen Z., Chen L.-x. (2016). Endoplasmic reticulum stress: a novel mechanism and therapeutic target for cardiovascular diseases. *Acta Pharmacologica Sinica*.

[4] Yang Y., Zhou Q., Gao A., Chen L., Li L. (2020). Endoplasmic reticulum stress and focused drug discovery in cardiovascular disease. *Clinica Chimica Acta*.

[5] Walter P., Ron D. (2011). The unfolded protein response: from stress pathway to homeostatic regulation. *Science*.

[6] Wang D., Yu W., Liu Y. (2017). Roles of autophagy in ischemic heart diseases and the modulatory effects of Chinese herbal medicine. *The American Journal of Chinese Medicine*.

[7] Wang D., Wang J., Liu Y., Zhao Z., Liu Q. (2016). Roles of Chinese herbal medicines in ischemic heart diseases (IHD) by regulating oxidative stress. *International Journal of Cardiology*.

[8] Wang J., Lin F., Guo L. L., Xiong X. J., Fan X. (2015). Cardiovascular disease, mitochondria, and traditional Chinese medicine. *Evidence-Based Complementary and Alternative Medicine*.

[9] Pan B., Sun J., Liu Z. (2021). Longxuetongluo capsule protects against cerebral ischemia/reperfusion injury through endoplasmic reticulum stress and MAPK-mediated mechanisms. *Journal of Advanced Research*.

[10] Schröder M., Kaufman R. J. (2005). The mammalian unfolded protein response. *Annual Review of Biochemistry*.

[11] Hetz C., Papa F. R. (2018). The unfolded protein response and cell fate control. *Molecular Cell*.

[12] Shen X., Ellis R. E., Sakaki K., Kaufman R. J. (2005). Genetic interactions due to constitutive and inducible gene regulation mediated by the unfolded protein response in C. elegans. *PLoS Genetics*.

[13] Bertolotti A., Wang X., Novoa I. (2001). Increased sensitivity to dextran sodium sulfate colitis in IRE1*β*-deficient mice. *Journal of Clinical Investigation*.

[14] So J. S. (2018). Roles of endoplasmic reticulum stress in immune responses. *Molecules and Cells*.

[15] Upton J.-P., Wang L., Han D. (2012). IRE1*α* cleaves select microRNAs during ER stress to derepress translation of proapoptotic caspase-2. *Science*.

[16] Maurel M., Chevet E., Tavernier J., Gerlo S. (2014). Getting RIDD of RNA: IRE1 in cell fate regulation. *Trends in Biochemical Sciences*.

[17] Shi Y., Vattem K. M., Sood R. (1998). Identification and characterization of pancreatic eukaryotic initiation factor 2*α*-subunit kinase, PEK, involved in translational control. *Molecular and Cellular Biology*.

[18] Bettigole S. E., Glimcher L. H. (2015). Endoplasmic reticulum stress in immunity. *Annual Review of Immunology*.

[19] Cullinan S. B., Zhang D., Hannink M., Arvisais E., Kaufman R. J., Diehl J. A. (2003). Nrf2 is a direct PERK substrate and effector of PERK-dependent cell survival. *Molecular and Cellular Biology*.

[20] Cullinan S. B., Diehl J. A. (2006). Coordination of ER and oxidative stress signaling: the PERK/Nrf2 signaling pathway. *The International Journal of Biochemistry & Cell Biology*.

[21] Li Y., Guo Y., Tang J., Jiang J., Chen Z. (2014). New insights into the roles of CHOP-induced apoptosis in ER stress. *Acta Biochimica et Biophysica Sinica*.

[22] Ma Y., Hendershot L. M. (2003). Delineation of a negative feedback regulatory loop that controls protein translation during endoplasmic reticulum stress. *Journal of Biological Chemistry*.

[23] Chen X., Shen J., Prywes R. (2002). The luminal domain of ATF6 senses endoplasmic reticulum (ER) stress and causes translocation of ATF6 from the ER to the golgi. *Journal of Biological Chemistry*.

[24] Thuerauf D. J., Marcinko M., Belmont P. J., Glembotski C. C. (2007). Effects of the isoform-specific characteristics of ATF6*α* and ATF6*β* on endoplasmic reticulum stress response gene expression and cell viability. *Journal of Biological Chemistry*.

[25] Ron D., Walter P. (2007). Signal integration in the endoplasmic reticulum unfolded protein response. *Nature Reviews Molecular Cell Biology*.

[26] Minamino T., Komuro I., Kitakaze M. (2010). Endoplasmic reticulum stress as a therapeutic target in cardiovascular disease. *Circulation Research*.

[27] Martindale J. J., Fernandez R., Thuerauf D. (2006). Endoplasmic reticulum stress gene induction and protection from ischemia/reperfusion injury in the hearts of transgenic mice with a tamoxifen-regulated form of ATF6. *Circulation Research*.

[28] Wang Z. V., Deng Y., Gao N. (2014). Spliced X-box binding protein 1 couples the unfolded protein response to hexosamine biosynthetic pathway. *Cell*.

[29] Ibuki T., Yamasaki Y., Mizuguchi H., Sokabe M. (2012). Protective effects of XBP1 against oxygen and glucose deprivation/reoxygenation injury in rat primary hippocampal neurons. *Neuroscience Letters*.

[30] Szegezdi E., Duffy A., O’Mahoney M. E. (2006). ER stress contributes to ischemia-induced cardiomyocyte apoptosis. *Biochemical and Biophysical Research Communications*.

[31] Lu P. D., Jousse C., Marciniak S. J. (2004). Cytoprotection by pre-emptive conditional phosphorylation of translation initiation factor 2. *The EMBO Journal*.

[32] Cullinan S. B., Diehl J. A. (2004). PERK-dependent activation of Nrf2 contributes to redox homeostasis and cell survival following endoplasmic reticulum stress. *Journal of Biological Chemistry*.

[33] Wang J., Lu L., Chen S. (2020). Up-regulation of PERK/Nrf2/HO-1 axis protects myocardial tissues of mice from damage triggered by ischemia-reperfusion through ameliorating endoplasmic reticulum stress. *Cardiovascular Diagnosis and Therapy*.

[34] Toth A., Nickson P., Mandl A., Bannister M., Toth K., Erhardt P. (2007). Endoplasmic reticulum stress as a novel therapeutic target in heart diseases. *Cardiovascular & Hematological Disorders-Drug Targets*.

[35] Miyazaki Y., Kaikita K., Endo M. (2011). C/EBP homologous protein deficiency attenuates myocardial reperfusion injury by inhibiting myocardial apoptosis and inflammation. *Arteriosclerosis, Thrombosis, and Vascular Biology*.

[36] Morse E., Schroth J., You Y.-H. (2010). TRB3 is stimulated in diabetic kidneys, regulated by the ER stress marker CHOP, and is a suppressor of podocyte MCP-1. *American Journal of Physiology—Renal Physiology*.

[37] Avery J., Etzion S., Debosch B. J. (2010). TRB3 function in cardiac endoplasmic reticulum stress. *Circulation Research*.

[38] Doroudgar S., Thuerauf D. J., Marcinko M. C., Belmont P. J., Glembotski C. C. (2009). Ischemia activates the ATF6 branch of the endoplasmic reticulum stress response. *Journal of Biological Chemistry*.

[39] Belmont P. J., Chen W. J., San Pedro M. N. (2010). Roles for endoplasmic reticulum-associated degradation and the novel endoplasmic reticulum stress response gene derlin-3 in the ischemic heart. *Circulation Research*.

[40] Jin J.-K., Blackwood E. A., Azizi K. (2017). ATF6 decreases myocardial ischemia/reperfusion damage and links ER stress and oxidative stress signaling pathways in the heart. *Circulation Research*.

[41] Paxman R., Plate L., Blackwood E. A. (2018). Pharmacologic ATF6 activating compounds are metabolically activated to selectively modify endoplasmic reticulum proteins. *eLife*.

[42] Blackwood E. A., Azizi K., Thuerauf D. J. (2019). Pharmacologic ATF6 activation confers global protection in widespread disease models by reprograming cellular proteostasis. *Nature Communications*.

[43] Liu X.-H., Zhang Z.-Y., Sun S., Wu X.-D. (2008). Ischemic postconditioning protects myocardium from ischemia/reperfusion injury through attenuating endoplasmic reticulum stress. *Shock*.

[44] Luo T., Kim J. K., Chen B., Abdel-Latif A., Kitakaze M., Yan L. (2015). Attenuation of ER stress prevents post-infarction-induced cardiac rupture and remodeling by modulating both cardiac apoptosis and fibrosis. *Chemico-Biological Interactions*.

[45] Kurian G. A., Rajagopal R., Vedantham S., Rajesh M. (2016). The role of oxidative stress in myocardial ischemia and reperfusion injury and remodeling. *Oxidative Medicine and Cellular Longevity*.

[46] Malhotra J. D., Kaufman R. J. (2007). Endoplasmic reticulum stress and oxidative stress: a vicious cycle or a double-edged sword?. *Antioxidants and Redox Signaling*.

[47] Tu B. P., Weissman J. S. (2004). Oxidative protein folding in eukaryotes. *Journal of Cell Biology*.

[48] Eletto D., Chevet E., Argon Y., Appenzeller-Herzog C. (2014). Redox controls UPR to control redox. *Journal of Cell Science*.

[49] Haynes C. M., Titus E. A., Cooper A. A. (2004). Degradation of misfolded proteins prevents ER-derived oxidative stress and cell death. *Molecular Cell*.

[50] Santos C. X. C., Nabeebaccus A. A., Shah A. M., Camargo L. L., Filho S. V., Lopes L. R. (2014). Endoplasmic reticulum stress and Nox-mediated reactive oxygen species signaling in the peripheral vasculature: potential role in hypertension. *Antioxidants and Redox Signaling*.

[51] Lassègue B., San Martín A., Griendling K. K. (2012). Biochemistry, physiology, and pathophysiology of NADPH oxidases in the cardiovascular system. *Circulation Research*.

[52] Li G., Scull C., Ozcan L., Tabas I. (2010). NADPH oxidase links endoplasmic reticulum stress, oxidative stress, and PKR activation to induce apoptosis. *Journal of Cell Biology*.

[53] Santos C. X. C., Tanaka L. Y., Wosniak J., Laurindo F. R. M. (2009). Mechanisms and implications of reactive oxygen species generation during the unfolded protein response: roles of endoplasmic reticulum oxidoreductases, mitochondrial electron transport, and NADPH oxidase. *Antioxidants and Redox Signaling*.

[54] Win S., Than T. A., Fernandez-Checa J. C., Kaplowitz N. (2014). JNK interaction with Sab mediates ER stress induced inhibition of mitochondrial respiration and cell death. *Cell Death & Disease*.

[55] Lerner A. G., Upton J.-P., Praveen P. V. K. (2012). IRE1*α* induces thioredoxin-interacting protein to activate the NLRP3 inflammasome and promote programmed cell death under irremediable ER stress. *Cell Metabolism*.

[56] Szabadkai G., Bianchi K., Várnai P. (2006). Chaperone-mediated coupling of endoplasmic reticulum and mitochondrial Ca2+ channels. *Journal of Cell Biology*.

[57] Bravo R., Gutierrez T., Paredes F. (2012). Endoplasmic reticulum: ER stress regulates mitochondrial bioenergetics. *The International Journal of Biochemistry & Cell Biology*.

[58] Van Vliet A. R., Agostinis P. (2018). Mitochondria-associated membranes and ER stress. *Current Topics in Microbiology and Immunology*.

[59] Rainbolt T. K., Saunders J. M., Wiseman R. L. (2014). Stress-responsive regulation of mitochondria through the ER unfolded protein response. *Trends in Endocrinology and Metabolism*.

[60] Anelli T., Bergamelli L., Margittai E. (2012). Ero1*α* regulates Ca2+ fluxes at the endoplasmic reticulum-mitochondria interface (MAM). *Antioxidants and Redox Signaling*.

[61] Kiviluoto S., Vervliet T., Ivanova H. (2013). Regulation of inositol 1,4,5-trisphosphate receptors during endoplasmic reticulum stress. *Biochimica et Biophysica Acta (BBA)—Molecular Cell Research*.

[62] St-Pierre J., Buckingham J. A., Roebuck S. J., Brand M. D. (2002). Topology of superoxide production from different sites in the mitochondrial electron transport chain. *Journal of Biological Chemistry*.

[63] Csordás G., Renken C., Várnai P. (2006). Structural and functional features and significance of the physical linkage between ER and mitochondria. *The Journal of Cell Biology*.

[64] Verfaillie T., Rubio N., Garg A. D. (2012). PERK is required at the ER-mitochondrial contact sites to convey apoptosis after ROS-based ER stress. *Cell Death & Differentiation*.

[65] Chaudhari N., Talwar P., Parimisetty A., Lefebvre d’Hellencourt C., Ravanan P. (2014). A molecular web: endoplasmic reticulum stress, inflammation, and oxidative stress. *Frontiers in Cellular Neuroscience*.

[66] Malhotra J. D., Miao H., Zhang K. (2008). Antioxidants reduce endoplasmic reticulum stress and improve protein secretion. *Proceedings of the National Academy of Sciences*.

[67] Peng T.-I., Jou M.-J. (2010). Oxidative stress caused by mitochondrial calcium overload. *Annals of the New York Academy of Sciences*.

[68] Bánsághi S., Golenár T., Madesh M. (2014). Isoform- and species-specific control of inositol 1,4,5-trisphosphate (IP3) receptors by reactive oxygen species. *Journal of Biological Chemistry*.

[69] Tabas I., Ron D. (2011). Integrating the mechanisms of apoptosis induced by endoplasmic reticulum stress. *Nature Cell Biology*.

[70] Li G., Mongillo M., Chin K.-T. (2009). Role of ERO1-*α*-mediated stimulation of inositol 1,4,5-triphosphate receptor activity in endoplasmic reticulum stress-induced apoptosis. *Journal of Cell Biology*.

[71] Timmins J. M., Ozcan L., Seimon T. A. (2009). Calcium/calmodulin-dependent protein kinase II links ER stress with Fas and mitochondrial apoptosis pathways. *Journal of Clinical Investigation*.

[72] Chami M., Oulès B., Szabadkai G., Tacine R., Rizzuto R., Paterlini-Bréchot P. (2008). Role of SERCA1 truncated isoform in the proapoptotic calcium transfer from ER to mitochondria during ER stress. *Molecular Cell*.

[73] Moore C. E., Omikorede O., Gomez E., Willars G. B., Herbert T. P. (2011). PERK activation at low glucose concentration is mediated by SERCA pump inhibition and confers preemptive cytoprotection to pancreatic *β*-cells. *Molecular Endocrinology*.

[74] Hayashi T., Su T.-P. (2007). Sigma-1 receptor chaperones at the ER- mitochondrion interface regulate Ca2+ signaling and cell survival. *Cell*.

[75] Son S. M., Byun J., Roh S.-E., Kim S. J., Mook-Jung I. (2014). Reduced IRE1*α* mediates apoptotic cell death by disrupting calcium homeostasis via the InsP3 receptor. *Cell Death & Disease*.

[76] Raturi A., Simmen T. (2013). Where the endoplasmic reticulum and the mitochondrion tie the knot: the mitochondria-associated membrane (MAM). *Biochimica et Biophysica Acta (BBA)—Molecular Cell Research*.

[77] Vitadello M., Penzo D., Petronilli V. (2003). Overexpression of the stress protein Grp94 reduces cardiomyocyte necrosis due to calcium overload and simulated ischemia. *FASEB Journal*.

[78] Zheng D., Wang G., Li S., Fan G.-C., Peng T. (2015). Calpain-1 induces endoplasmic reticulum stress in promoting cardiomyocyte apoptosis following hypoxia/reoxygenation. *Biochimica et Biophysica Acta—Molecular Basis of Disease*.

[79] Hotamisligil G. S. (2010). Endoplasmic reticulum stress and the inflammatory basis of metabolic disease. *Cell*.

[80] Kolattukudy P. E., Niu J. (2012). Inflammation, endoplasmic reticulum stress, autophagy, and the monocyte chemoattractant protein-1/CCR2 pathway. *Circulation Research*.

[81] Davis R. J. (2000). Signal transduction by the JNK group of MAP kinases. *Cell*.

[82] Montecucco F., Steffens S., Burger F., Pelli G., Monaco C., Mach F. (2008). C-reactive protein (CRP) induces chemokine secretion via CD11b/ICAM-1 interaction in human adherent monocytes. *Journal of Leukocyte Biology*.

[83] Yamazaki H., Hiramatsu N., Hayakawa K. (2009). Activation of the Akt-NF-*κ*B pathway by subtilase cytotoxin through the ATF6 branch of the unfolded protein response. *The Journal of Immunology*.

[84] Deng J., Lu P. D., Zhang Y. (2004). Translational repression mediates activation of nuclear factor kappa B by phosphorylated translation initiation factor 2. *Molecular and Cellular Biology*.

[85] Gotoh T., Endo M., Oike Y. (2011). Endoplasmic reticulum stress-related inflammation and cardiovascular diseases. *International Journal of Inflammation*.

[86] Azfer A., Niu J., Rogers L. M., Adamski F. M., Kolattukudy P. E. (2006). Activation of endoplasmic reticulum stress response during the development of ischemic heart disease. *American Journal of Physiology—Heart and Circulatory Physiology*.

[87] Cao L., Chen Y., Zhang Z., Li Y., Zhao P. (2019). Endoplasmic reticulum stress-induced NLRP1 inflammasome activation contributes to myocardial ischemia/reperfusion injury. *Shock*.

[88] Yi Y.-S. (2018). Role of inflammasomes in inflammatory autoimmune rheumatic diseases. *Korean Journal of Physiology and Pharmacology*.

[89] Zhang K., Shen X., Wu J. (2006). Endoplasmic reticulum stress activates cleavage of CREBH to induce a systemic inflammatory response. *Cell*.

[90] Xue X., Piao J.-H., Nakajima A. (2005). Tumor necrosis factor *α* (TNF*α*) induces the unfolded protein response (UPR) in a reactive oxygen species (ROS)-dependent fashion, and the UPR counteracts ROS accumulation by TNF*α*. *Journal of Biological Chemistry*.

[91] Yorimitsu T., Klionsky D. J. (2007). Endoplasmic reticulum stress: a new pathway to induce autophagy. *Autophagy*.

[92] Ding W.-X., Yin X.-M. (2008). Sorting, recognition and activation of the misfolded protein degradation pathways through macroautophagy and the proteasome. *Autophagy*.

[93] Ma S., Wang Y., Chen Y., Cao F. (2015). The role of the autophagy in myocardial ischemia/reperfusion injury. *Biochimica et Biophysica Acta—Molecular Basis of Disease*.

[94] Akao Y., Otsuki Y., Kataoka S., Ito Y., Tsujimoto Y. (1994). Multiple subcellular localization of bcl-2: detection in nuclear outer membrane, endoplasmic reticulum membrane, and mitochondrial membranes. *Cancer Research*.

[95] Liu K., Ren T., Huang Y. (2017). Apatinib promotes autophagy and apoptosis through VEGFR2/STAT3/BCL-2 signaling in osteosarcoma. *Cell Death & Disease*.

[96] Wei Y., Sinha S. C., Levine B. (2008). Dual role of JNK1-mediated phosphorylation of Bcl-2 in autophagy and apoptosis regulation. *Autophagy*.

[97] Rzymski T., Milani M., Singleton D. C., Harris A. L. (2009). Role of ATF4 in regulation of autophagy and resistance to drugs and hypoxia. *Cell Cycle*.

[98] Terai K., Hiramoto Y., Masaki M. (2005). AMP-activated protein kinase protects cardiomyocytes against hypoxic injury through attenuation of endoplasmic reticulum stress. *Molecular and Cellular Biology*.

[99] Wang X., Ron D. (1996). Stress-induced phosphorylation and activation of the transcription factor CHOP (GADD153) by p38 MAP kinase. *Science*.

[100] Bruhat A., Jousse C., Carraro V., Reimold A. M., Ferrara M., Fafournoux P. (2000). Amino acids control mammalian gene transcription: activating transcription factor 2 is essential for the amino acid responsiveness of the CHOP promoter. *Molecular and Cellular Biology*.

[101] Lu M., Lawrence D. A., Marsters S. (2014). Opposing unfolded-protein-response signals converge on death receptor 5 to control apoptosis. *Science*.

[102] Fu H. Y., Okada K.-i., Liao Y. (2010). Ablation of C/EBP homologous protein attenuates endoplasmic reticulum-mediated apoptosis and cardiac dysfunction induced by pressure overload. *Circulation*.

[103] Mccullough K. D., Martindale J. L., Klotz L.-O., Aw T.-Y., Holbrook N. J. (2001). Gadd153 sensitizes cells to endoplasmic reticulum stress by down-regulating Bcl2 and perturbing the cellular redox state. *Molecular and Cellular Biology*.

[104] Ghosh A. P., Klocke B. J., Ballestas M. E., Roth K. A. (2012). CHOP potentially co-operates with FOXO3a in neuronal cells to regulate PUMA and BIM expression in response to ER stress. *PLoS One*.

[105] Puthalakath H., O’Reilly L. A., Gunn P. (2007). ER stress triggers apoptosis by activating BH3-only protein Bim. *Cell*.

[106] Morishima N., Nakanishi K., Tsuchiya K., Shibata T., Seiwa E. (2004). Translocation of Bim to the endoplasmic reticulum (ER) mediates ER stress signaling for activation of caspase-12 during ER stress-induced apoptosis. *Journal of Biological Chemistry*.

[107] Scorrano L., Oakes S. A., Opferman J. T. (2003). BAX and BAK regulation of endoplasmic reticulum Ca2+: a control point for apoptosis. *Science*.

[108] Han J., Back S. H., Hur J. (2013). ER-stress-induced transcriptional regulation increases protein synthesis leading to cell death. *Nature Cell Biology*.

[109] Marciniak S. J., Yun C. Y., Oyadomari S. (2004). CHOP induces death by promoting protein synthesis and oxidation in the stressed endoplasmic reticulum. *Genes & Development*.

[110] Nashine S., Liu Y., Kim B.-J., Clark A. F., Pang I.-H. (2014). Role of C/EBP homologous protein in retinal ganglion cell death after ischemia/reperfusion injury. *Investigative Ophthalmology & Visual Science*.

[111] Gorman A. M., Healy S. J. M., Jäger R., Samali A. (2012). Stress management at the ER: regulators of ER stress-induced apoptosis. *Pharmacology & Therapeutics*.

[112] Nakagawa T., Zhu H., Morishima N. (2000). Caspase-12 mediates endoplasmic-reticulum-specific apoptosis and cytotoxicity by amyloid-*β*. *Nature*.

[113] Yoneda T., Imaizumi K., Oono K. (2001). Activation of caspase-12, an endoplastic reticulum (ER) resident caspase, through tumor necrosis factor receptor-associated factor 2-dependent mechanism in response to the ER stress. *Journal of Biological Chemistry*.

[114] Liu H., Wang Z., Nowicki M. J. (2014). Caspase-12 mediates carbon tetrachloride-induced hepatocyte apoptosis in mice. *World Journal of Gastroenterology*.

[115] Nakagawa T., Yuan J. (2000). Cross-talk between two cysteine protease families. *Journal of Cell Biology*.

[116] Hetz C. (2012). The unfolded protein response: controlling cell fate decisions under ER stress and beyond. *Nature Reviews Molecular Cell Biology*.

[117] Hata A. N., Engelman J. A., Faber A. C. (2015). The BCL2 family: key mediators of the apoptotic response to targeted anticancer therapeutics. *Cancer Discovery*.

[118] Thomenius M. J., Distelhorst C. W. (2003). Bcl-2 on the endoplasmic reticulum: protecting the mitochondria from a distance. *Journal of Cell Science*.

[119] Zong W.-X., Li C., Hatzivassiliou G. (2003). Bax and Bak can localize to the endoplasmic reticulum to initiate apoptosis. *Journal of Cell Biology*.

[120] Diwan A., Matkovich S. J., Yuan Q. (2009). Endoplasmic reticulum-mitochondria crosstalk in NIX-mediated murine cell death. *The Journal of clinical investigation*.

[121] Iurlaro R., Muñoz-Pinedo C. (2016). Cell death induced by endoplasmic reticulum stress. *FEBS Journal*.

[122] Kim H., Tu H.-C., Ren D. (2009). Stepwise activation of BAX and BAK by tBID, BIM, and PUMA initiates mitochondrial apoptosis. *Molecular Cell*.

[123] Hetz C., Bernasconi P., Fisher J. (2006). Proapoptotic BAX and BAK modulate the unfolded protein response by a direct interaction with IRE1*α*. *Science*.

[124] Nickson P., Toth A., Erhardt P. (2007). PUMA is critical for neonatal cardiomyocyte apoptosis induced by endoplasmic reticulum stress. *Cardiovascular Research*.

[125] Gerczuk P. Z., Breckenridge D. G., Liles J. T. (2012). An apoptosis signal-regulating kinase 1 inhibitor reduces cardiomyocyte apoptosis and infarct size in a rat ischemia-reperfusion model. *Journal of Cardiovascular Pharmacology*.

[126] Nishitoh H., Matsuzawa A., Tobiume K. (2002). ASK1 is essential for endoplasmic reticulum stress-induced neuronal cell death triggered by expanded polyglutamine repeats. *Genes & Development*.

[127] Demay Y., Perochon J., Szuplewski S., Mignotte B., Gaumer S. (2014). The PERK pathway independently triggers apoptosis and a Rac1/Slpr/JNK/Dilp8 signaling favoring tissue homeostasis in a chronic ER stress Drosophila model. *Cell Death & Disease*.

[128] Guo X., Meng Y., Sheng X. (2017). Tunicamycin enhances human colon cancer cells to TRAIL-induced apoptosis by JNK-CHOP-mediated DR5 upregulation and the inhibition of the EGFR pathway. *Anti-Cancer Drugs*.

[129] Fan M., Goodwin M., Vu T., Brantley-Finley C., Gaarde W. A., Chambers T. C. (2000). Vinblastine-induced phosphorylation of Bcl-2 and Bcl-XL is mediated by JNK and occurs in parallel with inactivation of the Raf-1/MEK/ERK cascade. *Journal of Biological Chemistry*.

[130] Malhi H., Kaufman R. J. (2011). Endoplasmic reticulum stress in liver disease. *Journal of Hepatology*.

[131] Zhao G.-l., Yu L.-m., Gao W.-l. (2016). Berberine protects rat heart from ischemia/reperfusion injury via activating JAK2/STAT3 signaling and attenuating endoplasmic reticulum stress. *Acta Pharmacologica Sinica*.

[132] Zhang W., Sun Z., Meng F. (2017). Schisandrin B ameliorates myocardial ischemia/reperfusion injury through attenuation of endoplasmic reticulum stress-induced apoptosis. *Inflammation*.

[133] Yu Y., Sun G., Luo Y. (2016). Cardioprotective effects of notoginsenoside R1 against ischemia/reperfusion injuries by regulating oxidative stress- and endoplasmic reticulum stress- related signaling pathways. *Scientific Reports*.

[134] Wei W., Peng J., LI J. (2019). Curcumin attenuates hypoxia/reoxygenationinduced myocardial injury. *Molecular Medicine Reports*.

[135] Wang C., Li Y.-Z., Wang X.-R., Lu Z.-R., Shi D.-Z., Liu X.-H. (2012). Panax quinquefolium saponins reduce myocardial hypoxia-reoxygenation injury by inhibiting excessive endoplasmic reticulum stress. *Shock*.

[136] Wan Y.-J., Wang Y.-H., Guo Q., Jiang Y., Tu P.-F., Zeng K.-W. (2021). Protocatechualdehyde protects oxygen-glucose deprivation/reoxygenation-induced myocardial injury via inhibiting PERK/ATF6*α*/IRE1*α* pathway. *European Journal of Pharmacology*.

[137] Sun M. Y., Ma D. S., Zhao S., Wang L., Ma C.-Y., Bai Y. (2018). Salidroside mitigates hypoxia/reoxygenation injury by alleviating endoplasmic reticulum stress induced apoptosis in H9c2 cardiomyocytes. *Molecular Medicine Reports*.

[138] Ge H., Guo Y., Zhou N., Yu Y. (2020). Astragaloside IV mediates SERCA 2a pathway to improve myocardial ischemia reperfusion injury in rats. *Progress of Anatomical Sciences*.

[139] Wang R., Yang M., Wang M. (2018). Total saponins of Aralia elata (Miq) seem alleviate calcium homeostasis imbalance and endoplasmic reticulum stress-related apoptosis induced by myocardial ischemia/reperfusion injury. *Cellular Physiology and Biochemistry*.

[140] Du Y., Wang M., Liu X. (2018). Araloside C prevents hypoxia/reoxygenation-induced endoplasmic reticulum stress via increasing heat shock protein 90 in H9c2 cardiomyocytes. *Frontiers in Pharmacology*.

[141] Wang M., Meng X.-b., Yu Y.-l. (2014). Elatoside C protects against hypoxia/reoxygenation-induced apoptosis in H9c2 cardiomyocytes through the reduction of endoplasmic reticulum stress partially depending on STAT3 activation. *Apoptosis*.

[142] Yu Y., Xing N., Xu X. (2019). Tournefolic acid B, derived from Clinopodium chinense (Benth.) Kuntze, protects against myocardial ischemia/reperfusion injury by inhibiting endoplasmic reticulum stress-regulated apoptosis via PI3K/AKT pathways. *Phytomedicine*.

[143] Bing S., Zhang R., Zhang X., Zhu S. (2020). Resveratrol alleviates myocardial ischemia-reperfusion injury in rats. *Journal of Shanxi Medical University*.

[144] Cui Y., Wang Y., Liu G. (2019). Protective effect of barbaloin in a rat model of myocardial ischemia reperfusion injury through the regulation of the CNPY2-PERK pathway. *International Journal of Molecular Medicine*.

[145] Imenshahidi M., Hosseinzadeh H. (2019). Berberine and barberry (Berberis vulgaris): a clinical review. *Phytotherapy Research*.

[146] Panossian A., Wikman G. (2008). Pharmacology of Schisandra chinensis Bail.: an overview of Russian research and uses in medicine. *Journal of Ethnopharmacology*.

[147] Nasser M. I., Zhu S., Chen C., Zhao M., Huang H., Zhu P. (2020). A comprehensive review on schisandrin B and its biological properties. *Oxidative Medicine and Cellular Longevity*.

[148] Pan C., Huo Y., An X. (2012). Panax notoginseng and its components decreased hypertension via stimulation of endothelial-dependent vessel dilatation. *Vascular Pharmacology*.

[149] Tong Q., Zhu P.-c., Zhuang Z. (2019). Notoginsenoside R1 for organs ischemia/reperfusion injury: a preclinical systematic review. *Frontiers in Pharmacology*.

[150] Kotha R. R., Luthria D. L. (2019). Curcumin: biological, pharmaceutical, nutraceutical, and analytical aspects. *Molecules*.

[151] Szczuka D., Nowak A., Zakłos-Szyda M. (2019). American ginseng (Panax quinquefolium L.) as a source of bioactive phytochemicals with pro-health properties. *Nutrients*.

[152] Wu W.-y., Wang Y.-p. (2012). Pharmacological actions and therapeutic applications of Salvia miltiorrhiza depside salt and its active components. *Acta Pharmacologica Sinica*.

[153] Gu M., Wang X., Su Z., Ouyang F. (2007). One-step separation and purification of 3,4-dihydroxyphenyllactic acid, salvianolic acid B and protocatechualdehyde from Salvia miltiorrhiza Bunge by high-speed counter-current chromatography. *Journal of Chromatography. A*.

[154] Guo C., Wang S., Duan J. (2017). Protocatechualdehyde protects against cerebral ischemia-reperfusion-induced oxidative injury via protein kinase c*ε*/nrf2/HO-1 pathway. *Molecular Neurobiology*.

[155] Zhu L., Wei T., Chang X. (2015). Effects of salidroside on myocardial injury in vivo in vitro via regulation of nox/NF-*κ*B/AP1 pathway. *Inflammation*.

[156] Zang Y., Wan J., Zhang Z., Huang S., Liu X., Zhang W. (2020). An updated role of astragaloside IV in heart failure. *Biomedicine & Pharmacotherapy*.

[157] Shikov A. N., Pozharitskaya O. N., Makarov V. G. (2016). Aralia elata var. mandshurica (Rupr. & Maxim.) J. Wen: an overview of pharmacological studies. *Phytomedicine*.

[158] Berman A. Y., Motechin R. A., Wiesenfeld M. Y., Holz M. K. (2017). The therapeutic potential of resveratrol: a review of clinical trials. *NPJ Precision Oncology*.

[159] Singab A.-N. B., El-Hefnawy H. M., Esmat A., Gad H. A., Nazeam J. A. (2015). A systemic review on Aloe arborescens pharmacological profile: biological activities and pilot clinical trials. *Phytotherapy Research*.

[160] Patel D., Patel K., Tahilyani V. (2012). Barbaloin: a concise report of its pharmacological and analytical aspects. *Asian Pacific Journal of Tropical Biomedicine*.

[161] Zhang Q., Shi J., Guo D. (2020). Qishen Granule alleviates endoplasmic reticulum stress-induced myocardial apoptosis through IRE-1-CRYAB pathway in myocardial ischemia. *Journal of Ethnopharmacology*.

[162] Wang R., Wang M., Zhou J. (2019). Shuxuening injection protects against myocardial ischemia-reperfusion injury through reducing oxidative stress, inflammation and thrombosis. *Annals of Translational Medicine*.

[163] Xia K., Wang Q., Li C., Zeng Z., Wang Y., Wang W. (2017). Effect of QSKL on MAPK and RhoA pathways in a rat model of heart failure. *Evidence-Based Complementary and Alternative Medicine*.

[164] Feng H., Chen X.-m., Li C.-y., Zhu R.-m., Fang J., Wang T.-y. (2012). Combined common femoral artery endarterectomy with superficial femoral artery stenting plus Shuxuening injection infusion for chronic lower extremity ischemia: 3-year results. *Chinese Journal of Integrative Medicine*.

[165] Wang S., Wang Z., Fan Q. (2016). Ginkgolide K protects the heart against endoplasmic reticulum stress injury by activating the inositol-requiring enzyme 1*α*/X box-binding protein-1 pathway. *British Journal of Pharmacology*.

